# Supplementary material for: Human T-cell leukemia virus type 1 may invalidate T-SPOT.TB assay results in rheumatoid arthritis patients: A retrospective case-control observational study
Source: PLoS One. 2020 May 27;15(5):e0233159. doi: 10.1371/journal.pone.0233159 (PMC7252607; doi:10.1371/journal.pone.0233159)
Supplement: S1 Table — (DOCX) [file pone.0233159.s001.docx]

|  | **Supplementary Table. Characteristics of HTLV-1-positive rheumatoid arthritis patients with between negative and invalid results of T-SPOT.*TB* assay** | | | | |
| --- | --- | --- | --- | --- | --- |
|  |  | | Negative results | Invalid results | p-value |
|  |  |  | (n= 12) | (n= 16) |  |
|  | Age, years (IQR) | | 70 (14.8) | 72 (9.3) | 0.83 |
|  | Female, no. (%) | | 9 (75) | 13 (81.3) | 0.69 |
|  |  | |  |  |  |
|  | Positive for RF, no. (%) ^a^ | | 9 (81.8) | 11 (68.7) | 0.45 |
|  | Positive for ACPA, no. (%) ^b^ | | 5 (71.4) | 9 (69.2) | 0.91 |
|  |  | |  |  |  |
|  | CRP (mg/dl) (IQR) | | 0.26 (1.1) | 0.11 (1.0) | 0.63 |
|  | ESR (mm/60min) (IQR) | | 17.5 (36.8) | 23 (33.8) | 0.64 |
|  | DAS28 (IQR) ^c^ | | 3.05 (1.6) | 2.84 (0.9) | 0.36 |
|  | CDAI (IQR) ^d^ | | 7.95 (9.5) | 3.55 (8.9) | 0.17 |
|  |  | |  |  |  |
|  | The number of white blood cells (/μl) (IQR) | | 6,921 (2,991) | 5,760 (1,800) | 0.52 |
|  | The number of lymphocytes (/μl) (IQR) | | 1,669 (1,074) | 1,694 (1,013) | 0.83 |
|  | Patients with corticosteroid treatment, no. (%) Dose of corticosteroid mg/day (IQR) ^e^ | | 7 (58.3) 6.3 (11.6) | 7 (43.7) 3 (3.2) | 0.74  0.22 |
|  | Patients with conventional DMARDs  (excluding MTX)  treatment, no. (%) | | 1 (8.3) | 4 (25) | 0.27 |
|  | Patients with MTX treatment, no. (%)  Dose of MTX mg/week (IQR) | | 4 (33.3) 9 (4.5) | 7 (43.7) 8 (4.0) | 0.57  0.75 |
|  | Patients with biologics treatment, no. (%) Monotherapy, no. (%) Combination with MTX, no (%) | | 5 (41.6) 4 / 5 (80) 1 / 5 (20) | 9 (51.7) 5 / 9 (55.5) 4 / 9 (44.5) | 0.44  0.37  0.37 |
|  |  | |  |  |  |
|  | Values are expressed as medians with interquartile range (IQR). Percentages (%) are calculated based on total number of patients in each group unless indicated otherwise. CRP, C-reactive protein; ESR, erythrosedimentation rate; DAS28, 28-Joint Disease Activity Score; CDAI, Clinical Disease Activity Index; DMARDs, disease-modifying anti-rheumatic drugs; MTX, methotrexate. ^a^ Data available in 11 and 16 patients of HTLV-1-positive RA patients with negative and invalid results groups, respectively.  ^b^ Data available in 7 and 13 patients of HTLV-1-positive RA patients with negative results and invalid results groups, respectively.  ^c^ Data available in 10 and 15 patients of HTLV-1-positive RA patients with negative results and invalid results groups, respectively. ^d^ Data available in 10 and 14 patients of HTLV-1-positive RA patients with negative results and invalid results groups, respectively.  ^e^ Prednisolone equivalent. | | | | |
|  |  |  | |  |  |
